# The complete chloroplast genome sequence of *Euphorbia lathyris* L. (Euphorbiaceae)

**DOI:** 10.1080/23802359.2020.1832601

**Published:** 2020-11-03

**Authors:** Wenqi Ma, Chaogeng Lv, Daiquan Jiang, Chuanzhi Kang, Dan Zhao

**Affiliations:** a National Resource Center for Chinese Meteria Medica, China Academy of Chinese Medical Sciences, Beijing, China; b School of Pharmacy, Guizhou University of Traditional Chinese Medicine, Guiyang, China

**Keywords:** *Euphorbia lathyris*, chloroplast genome, phylogenetic relationship

## Abstract

*Euphorbia lathyris* L. is a well-known bioenergy plant cultivated in many parts of the world. In this study, we sequenced the complete chloroplast (cp) genome sequence of *E. lathyris* to investigate its phylogenetic relationship in the family Euphorbiaceae. The cp genome was 163,738 bp in length, consisting of a pair of inverted repeats (IRa and IRb: 26,837 bp) separated by a large single-copy region (LSC: 91,783 bp) and a small single-copy region (SSC: 18,281 bp). The GC content of whole cp genome is 35.6%. Annotation showed the presence of 113 unique genes with 79 protein-coding genes, four tRNA genes, and 30 rRNA genes. Phylogenetic analysis indicated that *E. lathyris* was in the basal position of subgen. *Esula*, closely related to sect. *Esula* and sect. *Helioscopiae*.

*Euphorbia lathyris* L., or Caper spurge, is a very isolated species that is adventive in many parts of the world. It is probably native only in the Mediterranean region (Wu et al. 1994). The species is cultivated as an ornamental plant or developed as a commercial crop (Govaerts et al. 2000). With the gradual depletion of nonrenewable fossil energy and the rising oil price, people pay more attention to the development of bioenergy. *E. lathyris* is a kind of energy plant which can produce diesel oil. The oil content of its seeds is generally about 45%. All parts of the plant, including the seeds and roots, are poisonous. The seeds are used medicinally as a violent purgative in folk medicine.

Total genomic DNA from fresh leaves was extracted by using the method of Li et al. (2013). Voucher specimen of *E. lathyris* were collected from Li county, Hunan province, China(29°37′48″N, 111°45′36″E) and was stored at the herbarium of Institute of Chinese Materia Medica (CMMI), China Academy of Chinese Medical Sciences with the voucher number is 430723LY1047. The sequencing library was constructed and quantified following the methods introduced by Dong et al. (Dong et al. 2017; Sun et al. 2020). The whole genome sequencing was conducted with 150 bp paired-end reads on the Illumina HiSeq X Ten platform. Contigs were assembled from the high-quality paired-end reads by using the SPAdes 3.6.1 program (Kmer = 95) (Bankevich et al. 2012). The chloroplast genome contigs selected by the Blast program (Altschul et al. 1990), taken *Euphorbia esula* (GenBank: KY000001) as the reference. The selected contigs were assembled using Sequencher 4.10. Gene annotation of *E. lathyris* was performed using DOGMA annotation (Wyman et al. 2004) and manually corrected for codons and gene boundaries using BLAST searches. The annotated cp genome of *E. lathyris* was submitted to the GenBank under the accession number MT830859.

The circular cpDNA of *E. lathyris* was 163,738 bp in length, consisting of a pair of inverted repeats (IRa and IRb: 26,837 bp) separated by a large single-copy region (LSC: 91,783 bp) and a small single-copy region (SSC: 18,281 bp). The GC content of whole cp genome is 35.6%. The cpDNA of *E. lathyris* comprised 113 distinct genes, including 79 protein-coding genes, four ribosomal RNA genes, and 30 transfer RNA genes. In these genes, 19 were duplicated in the IR regions and 19 genes contained one or two introns, 17 harbored a single intron, and two (*ycf3*,*clpP*) contained double introns.

To investigate the phylogenetic relationships between *E. lathyris* and other related species in family Euphorbiaceae, Twenty chloroplast genome sequences were downloaded from GenBank to construct a phylogenetic IQ-tree using PhyloSuite under the TVM + F + I + G4 model with 1000 bootstrap replicates (Nguyen et al. 2015; Zhang et al. 2020). *Daphniphyllum oldhamii* and *D. macropodum* were taken as outgroups. The phylogenetic analysis revealed that samples of genus *Euphorbia* were strongly supported as monophyletic, *E. lathyris* was in the basal position of subgen. *Esula* additionally (Figure 1). The cpDNA of *E. lathyris* is closly related to sect. *Esula* and sect. *Helioscopiae*. The complete chloroplast genome reported in this study will be a valuable resource for future studies on genetic diversity, taxonomy, and phylogeny of family Euphorbiaceae.

**Figure 1.**
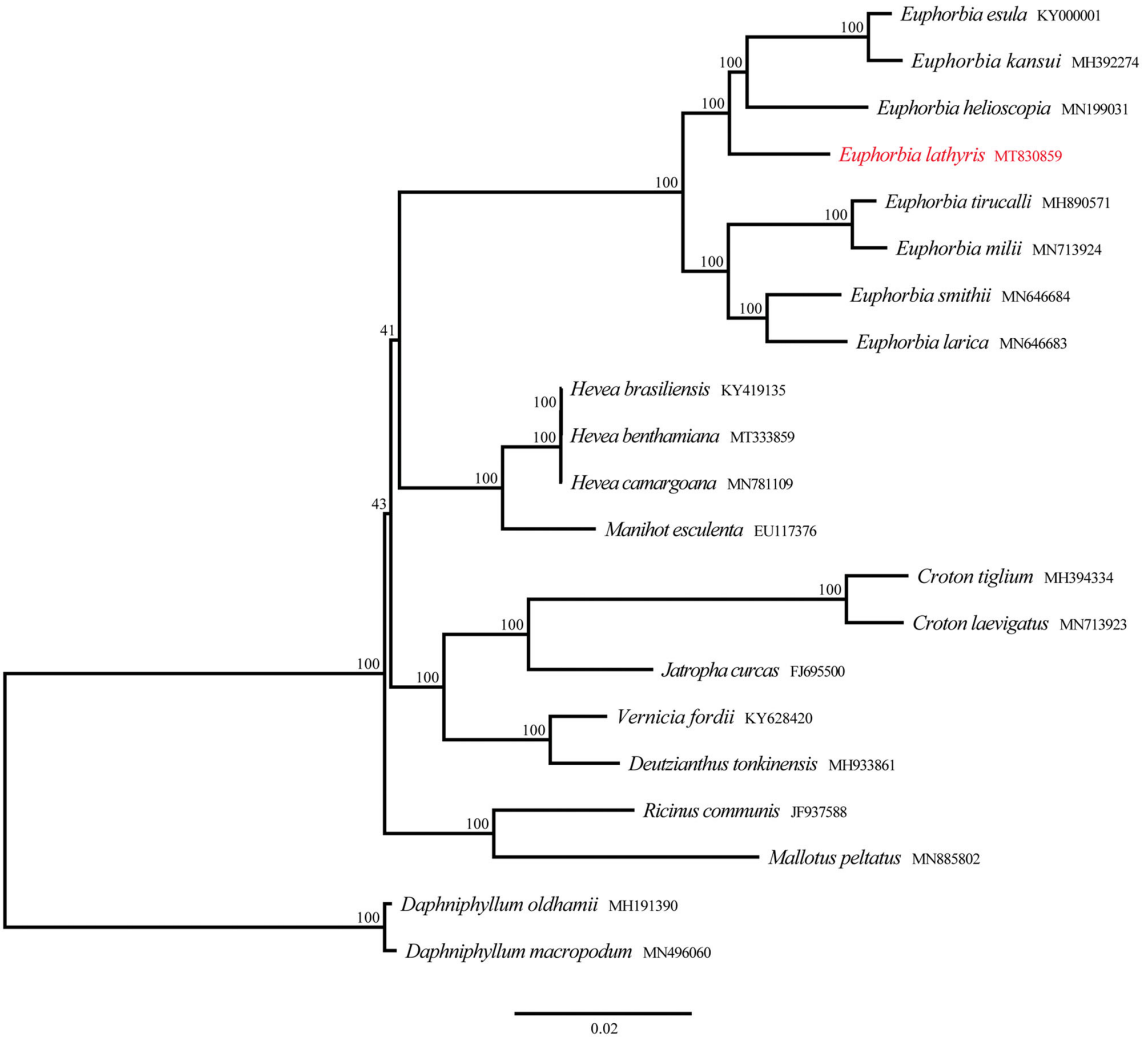
Phylogenetic tree reconstruction of 21 taxa using maximum likelihood (ML) methods in the chloroplast genome sequences. ML bootstrap support value is presented at each node.

## Data Availability

The data that support the findings of this study are openly available in GenBank of NCBI https://www.ncbi.nlm.nih.gov/, reference number MT830859, raw data BioProject ID: PRJNA660005, Biosample (SAMN15932209).
